# Population-Based Childhood Overweight Prevention: Outcomes of the ‘Be Active, Eat Right’ Study

**DOI:** 10.1371/journal.pone.0065376

**Published:** 2013-05-31

**Authors:** Amy van Grieken, Lydian Veldhuis, Carry M. Renders, Gerard J. Borsboom, Johannes C. van der Wouden, Remy A. Hirasing, Hein Raat

**Affiliations:** 1 Department of Public Health, Erasmus MC University Medical Center Rotterdam, Rotterdam, The Netherlands; 2 Department of Health Sciences, Faculty of Earth and Life Sciences, VU University Amsterdam, Amsterdam, The Netherlands; 3 EMGO Institute for Health and Care Research, VU University Amsterdam, Amsterdam, The Netherlands; 4 Department of General Practice, Erasmus MC, University Medical Center, Rotterdam, The Netherlands; 5 Department of General Practice and Elderly Care Medicine, VU University Medical Center, Amsterdam, The Netherlands; 6 Department of Public and Occupational Health, VU University Medical Center, Amsterdam, The Netherlands; NIDDK/NIH, United States of America

## Abstract

**Objective:**

An overweight prevention protocol was used in the ‘Be active, eat right’ study; parents of overweight children (5 years) were offered healthy lifestyle counseling by youth health care professionals. Effects of the protocol on child BMI and waist circumference at age 7 years were evaluated.

**Methods:**

A cluster RCT was conducted among nine youth health care centers in the Netherlands. Parents of overweight, not obese, children received lifestyle counseling and motivational interviewing according to the overweight prevention protocol in the intervention condition (n = 349) and usual care in the control condition (n = 288). Measurements were made of child height, weight and waist circumference at baseline and at a two-year follow-up; parents completed questionnaires regarding demographic characteristics. Linear mixed models were applied; interaction terms were explored.

**Results:**

The analyzed population consisted of 38.1% boys; mean age 5.7 [sd: 0.4] years; mean BMI 18.1 [sd: 0.6], the median number of counseling sessions in the intervention condition was 2. The regression model showed no significant difference in BMI increase between the research conditions at follow-up (beta −0.16; 95% CI:−0.60 to 0.27; p = 0.463). There was a significant interaction between baseline BMI and research condition; children with a baseline BMI of 17.25 and 17.50 had a smaller increase in BMI at follow-up when allocated to the intervention condition compared to control condition (estimated adjusted mean difference −0.67 [se: 0.30] and −0.52 [se: 0.36]).

**Conclusion:**

Mildly overweight children (baseline BMI 17.25 and 17.50) in the intervention condition showed a significantly smaller increase in BMI at follow-up compared to the control condition; there was no overall difference between intervention and control condition. Future research may explore and evaluate improvements of the prevention protocol.

**Trial Registration:**

Current Controlled Trials ISRCTN04965410

## Introduction

The prevalence of childhood overweight and obesity has been increasing for several years [1]. In the Netherlands in 2009, the prevalence of overweight among boys was estimated at 12.8% and obesity at 1.8%, while for girls the figures were 14.8% and 2.2%, respectively (2–21 years) [2]. Several consequences are associated with overweight, and especially obesity, in childhood (e.g., type 2 diabetes, heart disease) [3], [4]. Worldwide, interventions aimed at preventing overweight and obesity among children are being developed and evaluated [5], [6]. It has been shown that parental involvement may contribute to improving healthy behavior in children and preventing the development of overweight and obesity [7], [8], [9].

In the Netherlands, growth, development and health of all children (0–19 years) is monitored in a nationwide program with regular well-child visits at set ages by providers of preventive Youth Health Care (YHC). In each Dutch region YHC providers, mainly pediatricians and school-nurses, work in teams at YHC centers or schools to execute this nationwide program [10], [11]. The nationwide program is offered free of charge by the government; participation is voluntary (attendance rate 90–100%) [12]. Several successful preventive measures have been implemented through the YHC, for example the national immunization program and the prevention of Sudden Infant Death Syndrome (SIDS) [13], [14]. The YHC setting with the regular visits, measurement of height and weight and also the collaboration between YHC providers and local care providers, creates an opportunity for tailored prevention and promotion of healthy child development.

In 2004, a practiced-based protocol was developed to help detect overweight and obesity among children attending a well-child visit [15], [16]. Children were by means of this protocol classified into weight categories using the international age-and-gender Body Mass Index (BMI) cut-off values [17]. In 2005, a transitional plan, the prevention protocol, was developed based on the international literature and theory, to be used in daily practice to prevent overweight children from developing obesity [18]. This intervention offers additional healthy lifestyle counseling to parents of overweight, not obese children [18]. During a well-child visit, parents are informed about the overweight of their child and motivated to change health behavior. In addition, up to three structured healthy lifestyle counseling sessions to promote overweight-prevention behaviors can be offered. The YHC professionals are trained in using motivational interviewing to motivate parents to change health behavior, taking into account the parents' current stage of change [18].

The prevention protocol was launched as a transitional plan in 2005. The “Be active eat right” study was set up to assess the effectiveness of this prevention protocol among 5-year-old children who are overweight (not obese)[19]. We hypothesized that the children who are overweight (not obese) visiting YHC teams allocated to the intervention condition, selected to perform the prevention protocol, would have a less increase in BMI and waist circumference at follow-up compared to overweight children visiting YHC teams allocated to the control condition, performing usual care.

## Methods

The ‘Be active, eat right’ study (trial registration Current Controlled Trials ISRCTN04965410) is a cluster randomized controlled trial described in detail elsewhere [19]. In 2007 all YHC centers in the Netherlands (n = 37) were invited to participate in the study. Nine centers were eligible (i.e. a control condition could be created) and agreed to participate with a total of 44 YHC teams [19]. Within each center, YHC teams were randomized for allocation to intervention or control condition by means of a computer-generated random number list. All parents and children are invited for a well-child visit in the year the child turns five years old. Between September 2007 and October 2008 all parents invited to attend the well-child visit of their five-year-old child were also invited to participate in the study with their children. Information on the study and an informed consent form for participation in the two-year study was enclosed with the invitation for the well-child visit. Parents were requested to complete the informed consent form and hand it in the at the start of the well-child visit; parents provided written informed consent on behalf of themselves and their child for participation in the two-year study. Parents and children participated for two subsequent years, from the well-child visit onwards; two-year follow-up assessments were performed from September 2009 until October 2010.

The Medical Ethics Committee of the Erasmus University Medical Center Rotterdam approved the study protocol (reference number MEC-2007-163). Parents were not aware of the research condition they were allocated to. The protocol for this trial and supporting CONSORT checklist are available as supporting information; see Checklist S1 and Protocol S1.

### Intervention

When parent and child attended the well-child visit at a YHC team allocated to the intervention condition, and the child was classified as being overweight (not obese), the parents were offered the prevention protocol [17], [18]. The prevention protocol offered parents information regarding overweight prevention and healthy lifestyle choices by using a motivational interviewing approach, if needed, to motivate the parents to change behavior [20]. The prevention protocol was initiated during the well-child visit and in addition up to three structured healthy lifestyle counseling sessions to promote overweight-prevention behaviors could be offered, approximately 3, 6 and 12 months after the well-child visit.

The content of an additional counseling session depended on the stage of behavioral change of the parents [19], [21], [22]. The YHC professionals assessed the level of motivation of the parent during the well-child visit. The YHC professionals needed to create awareness of the child's weight status among the parents. Information about overweight and associated consequences could be given to parents. Moreover, motivational interviewing techniques could be used to further motivate parents to change health behavior. The four lifestyle-related behaviors that are described in the protocol and could be promoted were playing outdoors and eating breakfast, and reducing sweet drinks and sedentary behavior (watching television, computer gaming). Parents together with YHC professionals choose one or two behaviors to target during the sessions. Advice to parents was given according to international guidelines (play outside at least one hour a day, have breakfast daily, drink no more than 2 glasses of sweet beverages and limit sedentary behavior to maximum of 2 hours a day) [18], [22]. Information materials were provided to parents during the session, diet and activity diaries were discussed and family-oriented action plans to change health-related behavior were documented.

When a child who was overweight (not obese) was detected when visiting a YHC team allocated to the control condition, parents were also informed about the overweight of their child but usual care was given. Usual care consisted of general information about a healthy lifestyle during the well-child visit.

In accordance with the protocol, all YHC professionals, regardless of their team allocation, had to refer obese children to the general practitioner for further diagnosis and management. In the overweight prevention protocol, no additional care is prescribed for normal-weight and underweight children.

### Outcome measurements

Data collection was scheduled at enrollment, baseline (the well-child visit), and two years post-baseline (follow-up). Parents received a questionnaire enclosed with the invitation for the well-child visit. The parents could return the questionnaire during the well-child visit when their child was five years old. Two years after the well-child visit, parents received an invitation for a second measurement of their child's height, weight and waist circumference and a questionnaire, which could be completed on paper or via the Internet.

At baseline, YHC professionals at the YHC center measured the height, weight and waist circumference of all children. At age 7 years, follow-up, there was no regular visit planned with the YHC professionals. At two centers, the YHC professionals were able to perform the measures, but in all other regions research assistants performed the measures visiting the children's primary schools. Both YHC professionals and research assistants used the same standardized methods and equipment as described in a protocol [16]. Research assistants were blinded to the research condition at the time of follow-up evaluation.

### Primary outcomes

The BMI and waist circumference at follow-up were the primary outcome measurements, both were measured according to national protocols. Body weight was measured to the nearest 0.1 kilogram and height to the nearest 0.1 centimeter. Waist circumference was measured over the naked skin at the level midway between the lower rib margin and the iliac crest at the end of gentle expiration, while the children were in standing position [16]. The data collectors were trained to measure waist circumference using a measuring tape (SECA 200). BMI was calculated by dividing weight in kilogram by height per meter squared. Children were classified into having normal weight, overweight (not obesity) or obesity according to the international age and gender specific cut-off points for BMI [23].

### Other measures

Information on the child's age (in months) was obtained from the well-child visit registration. Information on the child's gender (male, female) and ethnic background (Dutch, non-Dutch) was obtained at enrollment via the parent report. Child ethnic background was categorized according to the parents' country of birth: if both parents were born in the Netherlands the child was classified “Dutch,” otherwise the child was classified “non-Dutch” [24].

The majority of the questionnaires were completed by mothers (88.1%). Information on maternal age (years), height (meters), weight (kilograms), country of birth (the Netherlands, other countries) and educational level (low/mid-low, mid-high/high) was self-reported in the baseline questionnaire. Maternal BMI was calculated and dichotomized into normal weight (BMI<25 kg/m^2^) or overweight (BMI≥25 kg/m^2^) [25]. Maternal education level was dichotomized: low/mid-low (no education, primary school, or ≤3 years of general secondary school, >3 years of general secondary school) or mid-high/high (higher vocational training, undergraduate programs, Bachelor's degree, higher academic education) [26].

The YHC professionals in both intervention and control condition were to return a registration form after the well-child visit. The YHC professionals in the intervention condition also returned a registration form after each additional session. The forms addressed session duration, topics discussed, whether action plans for change or workbook-exercises were discussed, and whether a new session was planned. If there was no follow-up session planned, the reasons therefore were recorded. Questionnaires assessing the acceptability and feasibility of the prevention protocol were sent to the parents and YHC professionals after the first or second additional session. The YHC professionals could indicate challenges of the prevention protocol and give an overall grade. Parents were asked to indicate whether the information provided during the sessions was appreciated.

### Sample size considerations

The calculation for the sample size in this study is described elsewhere [19]. The sample size calculations were based on an expected intra-cluster correlation coefficient (ICC) of 0.1, n = 44 clusters, an expected prevalence of overweight of 9%, while the power of the study was set to 80%. Based on these assumptions, a difference of 0.35 kg/m^2^, assuming a standard deviation of 1.0 kg/m^2^, could be detected, if the final sample of overweight children consisted of n = 356 subjects (n = 178 in each research condition).

The observed ICC for follow-up BMI and waist circumference was calculated. A regression model with only a fixed intercept and a random intercept for YHC team was fitted; no other variables were included. Covariance parameters were used to calculate the ICC as cluster variance/total variance. The observed ICC for BMI and waist circumference were rho = 0.06 and rho = 0.11 respectively. In the analyses, the regression models predicting follow-up BMI and waist circumference, included a random intercept for YHC team to correct for clustering.

### Statistical analysis

Baseline data for the intervention and control condition clusters are described using descriptive statistics.

To predict follow-up BMI and waist circumference, regression models were applied. All participants were analyzed according to the “intention-to-treat” principle. At follow-up, the population for BMI analysis was n = 505 and for waist circumference n = 482.

BMI and waist circumference at follow-up were predicted with a model using two predictors: research condition (intervention or control) and baseline value of the outcome variable [28], [29]. Time between baseline and follow-up measurement was added to the model (mean 26.0 [sd: 4.42] months, range 14 to 35 months, mean intervention condition 26.08 [sd: 4.48], mean control condition 25.91 [sd:4.36]). A sensitivity analysis was conducted to compare the final model with and without inclusion of time between measurements; results were similar. Age at the baseline measurement was added to the model (mean 69.6 [sd: 5.18] months, range 56.4 to 91.2). Gender and ethnic background of the child, and education level and BMI of the mother were evaluated as potential confounding variables. All models are presented with and without correction for clustering at YHC team-level (n = 44) [27]. The effect of the intervention was evaluated at p<0.05 level in all analyses.

Interaction effects between the outcome variable and socio-demographic characteristics (gender and ethnic background of the child, education level of the mother) were examined [19], [30], [31]. Additionally, a post-hoc analysis was performed and an interaction term between research condition and baseline value of the outcome was evaluated. Both a main effect for the interaction variable and an effect for the interaction term –research condition times interaction variable– were added to the regression model. The interaction terms were evaluated at p<0.10 level [32].

In addition, the regression analyses were performed using age- and gender adjusted BMI-SDS scores based on the reference values of the in 1997 performed Dutch nation-wide growth study [33]. A per protocol analysis was performed comparing overweight children of parents that attended at least one additional session with overweight children in the control condition. Process measures with regard to use and appreciation of the intervention are described using descriptive statistics. Non-response to the intervention was evaluated by comparing mothers of children who attended at least one additional session with mothers that did not attend any additional session on demographical characteristics age, country of birth, education level and BMI.

Analyses were performed in SPSS (International Business Machines (IBM) Corp., SPSS statistics, version 20.0, Armonk, New York, USA). Cluster adjusted regression models were fitted using SAS software; proc mixed for continues outcomes with a random intercept for YHC team (SAS version 9.2; SAS Institute Inc., Cary, North Carolina, USA).

## Results

**Figure 1:**
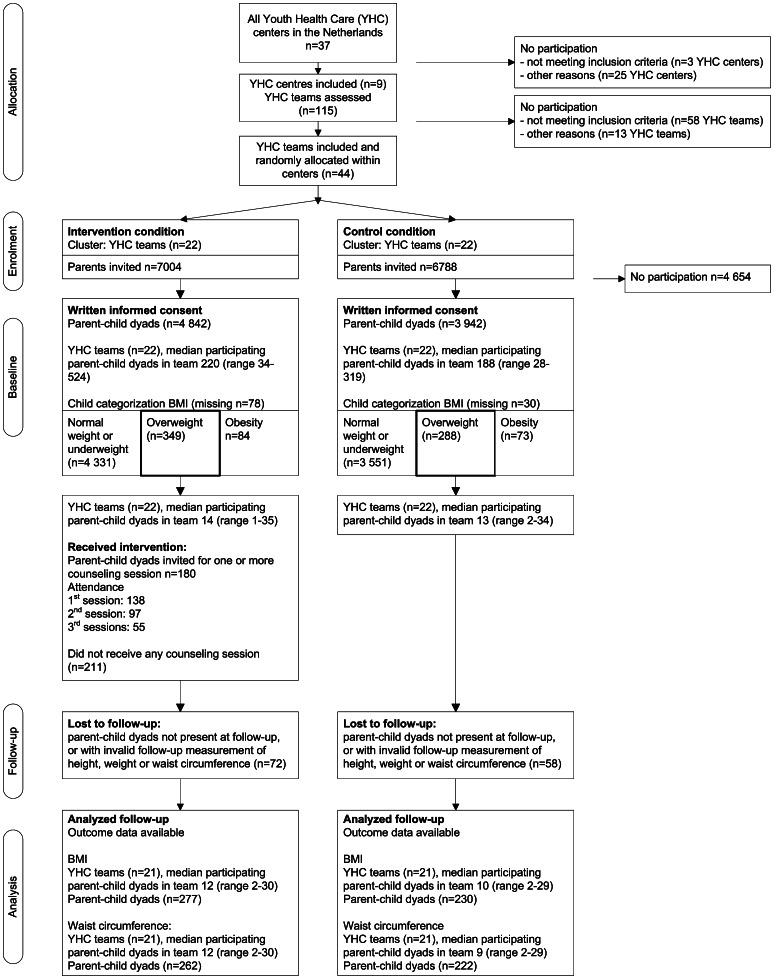
Flow diagram of the selection and follow-up of study participants.

Figure 1 presents the flow of clusters and participants through the study. In order to detect children who are overweight a total of 13 638 parents and children was invited to participate in the study; 8 784 parents agreed to participate and provided written informed consent (64.4%) (for descriptive characteristics of all study participants with informed consent please see Table S1). Of these parents, 637 had a child that was overweight, not obese (7.3%).

In Table 1 characteristics of the children who were overweight (not obese) at baseline are presented; 38.1% were boys, the mean age at baseline was 69.09 [sd: 5.18] months. Baseline BMI ranged from 17.5 (age: 63.0 months) to 19.8 (age: 78.0 months) in boys and from 17.2 (age: 61.0 months) to 19.9 (age: 77.0 months) in girls.

**Table 1:** Descriptive characteristics of the study sample, overweight not obese children (n = 637).

	Overall (n = 637)	Intervention condition (n = 349)	Control condition (n = 288)	*p-value* *
**Child characteristics**				
Age, mean (sd), months (missing n = 0)	69.09 (5.18)	68.65 (4.98)	69.64 (5.37)	**0.016**
Gender (% boys) (missing n = 0)	38.1	38.7	37.5	0.412
Ethnic background (% Dutch) (missing n = 11)	78.0	75.8	80.6	0.091
BMI, mean (sd), kg/m^2^ (missing n = 0)	18.13 (0.62)	18.16 (0.63)	18.10 (0.61)	0.238
**Mothers' characteristics**				
Age, mean (sd), years (missing n = 81)	35.85 (4.29)	35.80 (4.23)	35.92 (4.37)	0.741
Country of birth (% the Netherlands) (missing n = 4)	83.1	82.4	84.0	0.335
Education level (missing n = 6)				0.214
Low/Mid-low	33.3	34.8	31.5	
Mid-high/High	66.7	65.2	68.5	
BMI categories (missing n = 51)				0.422
Normal weight	56.0	55.5	56.6	
Overweight/obesity	44.0	44.5	43.4	

* P-value derived from independent samples t-test (continuous variables) Chi Square test (categorical variables).

Note: **bold** printed numbers indicate significant p-value.

### Primary outcomes

At baseline child overweight prevalence was 7.3% in the intervention condition and 7.4% in the control condition (missing n = 108, see Figure 1). At follow-up the prevalence of normal weight, overweight and obesity was 87.2%, 10.8% and 2.1% in the intervention condition and 86.4%, 11.4% and 2.2% in the control condition respectively (missing n = 1 693).

Of the children in the intervention condition that were overweight at baseline, at follow-up 61.0% remained overweight, 14.4% were categorized as obese and 24.5% normal weight. In the control condition among the children that were overweight at baseline, the prevalence of overweight, obesity and normal weight was 62.7%, 11.0% and 26.3% respectively at follow-up (missing n = 60).

The mean change in BMI from baseline to follow-up was 1.37 [sd: 1.53] in the intervention condition versus 1.44 [sd: 1.71] in the control condition. The regression model showed that, at follow-up, there was no significant difference in BMI between children in the intervention condition compared to the control condition (19.53 [sd: 1.72] versus 19.55 [sd: 1.74], beta intervention condition −0.16; 95% CI: −0.60 to 0.27, p = 0.46) (Table 2). In the model predicting BMI, there was a statistically significant interaction between research condition and baseline BMI (p = 0.01) (Table 2). This indicated that the overall slopes of the regression lines for intervention condition and control condition were not equal (please see Figure S1 for a graphical representation of this regression model). To gain more insight in the observed interaction we additionally evaluated for which exact value of the baseline BMI the difference between the intervention and control condition follow-up BMI was statistically significant. To explore this, marginal mean differences were estimated for baseline BMI values (17.25 to 19.25 with intervals of 0.25 kg/m^2^) (Table 3). The results indicate that at the baseline BMI values of 17.25 and 17.50, the intervention and control condition had a significant difference in mean follow-up BMI (adjusted estimated mean difference −0.67 [se: 0.30], p = 0.02 and −0.52 [se: 0.36], p = 0.05) (Table 3).

**Table 2:** Outcomes of linear regression models predicting BMI (kg/m^2^) and waist circumference (cm) at follow-up.

	BMI (n = 505)		Waist circumference (n = 482)	
	Beta coefficient (95%CI)	*p-value*	Beta coefficient (95%CI)	*p-value*
Model 1^1^	−0.06 (−0.34; 0.23)	0.687	−0.16 (−1.10; 0.78)	0.741
Model 2^2^	−10.25 (−18.57; −1.93)	**0.016**	10.04 (−4.94; 25.01)	0.188
Interaction term	0.56 (0.10; 1.02)	**0.016**	−0.17 (−0.43; 0.08)	0.181
Model 3^3^	−0.16 (−0.60; 0.27)	0.463	−0.46 (−1.82; 0.90)	0.506
Model 4^4^	−10.67 (−18.80; −2.54)	**0.010**	7.98 (−7.39; 23.34)	0.308
Interaction term	0.58 (0.13; 1.03)	**0.011**	−0.14 (−0.40; 0.12)	0.280

1 Model with research condition (intervention vs. control condition), baseline value of the outcome variable, time between measurements and age of the child at baseline measurement.

2 Model 1 including an interaction term between baseline BMI (continuous) and research condition (intervention condition compared to control condition – reference).

3 Model 1 corrected for cluster.

4 Model 3 including an interaction term between baseline BMI (continuous) and research condition (intervention condition compared to control condition – reference).

Note: **bold** printed numbers indicate significant p-value.

**Table 3:** Estimated least square marginal mean difference between intervention and control condition for given baseline BMI values.

Baseline BMI (kg/m^2^)	Estimated adjusted difference (se) between intervention condition and control condition^1^ *	*p-value*
**17.25**	**−0.67 (0.30)**	**0.024**
**17.50**	**−0.52 (0.26)**	**0.045**
17.75	−0.38 (0.23)	0.106
18.00	−0.24 (0.22)	0.286
18.25	−0.09 (0.22)	0.382
18.50	0.05 (0.23)	0.815
18.75	0.20 (0.26)	0.443
19.00	0.34 (0.29)	0.243
19.25	0.49 (0.34)	0.146
19.50	0.63 (0.38)	0.096

1 Model is corrected for cluster with research condition (intervention vs. control condition), baseline value of the outcome variable, time between measurements and age of the child at baseline as independent predictors; model includes an interaction between baseline BMI and research condition.

* Covariate “time between measurements” was evaluated in the model at the mean of 26.0 months and age at baseline was evaluated at the mean of 69.0 months.

Note: **bold** printed numbers indicate a significant estimated adjusted difference between intervention and control condition.

The mean change in waist circumference from baseline to follow-up was 7.20 [sd: 5.49] centimeter in the intervention condition and 7.33 [sd: 5.30] centimeter in the control condition. At follow-up children in the intervention condition had a waist circumference of 65.60 [sd: 6.07] centimeter versus 66.21 [sd: 6.03] centimeter in the control condition (beta intervention condition −0.46 centimeter; 95% CI: −1.82 to 0.90, p = 0.51). There were no statistically significant interaction terms (Table 2).

The regression analyses performed with BMI-SDS scores (data not shown) also showed a significant interaction term for intervention condition times baseline BMI-SDS score (p = 0.07); indicating similar results as the analyses with the BMI scores. The per protocol analysis (data not shown), comparing children from parents attending at least one additional session (n = 138) with parents in the control condition (n = 288), showed similar results as the intention to treat analysis.

### Evaluation of the intervention

The YHC professionals performing the well-child visit were mainly pediatricians (72.0%). The YHC professionals did not invite all overweight children and parents for an additional session (51.6%, 180/349). The main reason for not inviting parents for an initial session was that based on the YHC professional judgment, the child was not overweight (n = 73) because of differences in posture, ethnicity or body composition. Other reasons were that the YHC professional was not able to motivate the parents or the parents refused additional counseling (n = 35) or the child had other problems that had priority, such as behavioral problems (n = 15).

Additional sessions were mainly performed by pediatricians (65.8%). Attendance at the first session was 76.7% (138/180), the second session 53.9% (97/180), and all three sessions 30.6% (55/180) (Figure 1). Average duration of first additional session was 24.76 minutes [sd: 10.51, range 0–60 minutes]. The baseline BMI of children whose parents attended at least one additional session was higher compared to the BMI of children whose parents did not attend any additional session (mean BMI 18.30 [sd: 0.59] versus 18.07 [sd: 0.64], p = 0.001). Mothers that attended at least one session (n = 138) were not statistically different with regard to age, country of birth, education level or BMI from mothers that did not attend any additional session.

The YHC professionals in the intervention condition filled in evaluation forms on the use of the prevention protocol (n = 54). In the intervention teams, 65% (15/23, n = 31 missing) of the YHC professionals evaluated the prevention protocol with a grade of 7 or higher (scale range 1 to 10). Difficulties the YHC professionals most often experienced while using the prevention protocol were motivating parents to attend additional sessions and changing the family health-related lifestyle.

The additional sessions were assessed with a grade of 7 or higher by 90% (81/90) of the parents that filled in an evaluation form; 87% (78/90) reported receiving overall useful information, and 79% (71/90) reported receiving advice that suited them.

## Discussion

In this study, the overweight prevention protocol was evaluated for parents of five-year-old overweight (not obese) children. Results showed no overall difference between children in the intervention and control condition with regard to BMI and waist circumference at two-year follow-up. However, a significant interaction effect was found when predicting follow-up BMI, indicating that children with a relatively low overweight BMI (17.25 and 17.50) at the start of the intervention had a smaller increase in BMI at follow-up when they had been allocated to the intervention condition relative to the control condition. This interaction was also observed when performing analysis based on BMI-SDS scores and with a per protocol analysis comparing overweight children of parents that attended at least one additional session to overweight children in the control condition.

The intervention showed a small effect among the mildly overweight children, but not for the more overweight children. Specifically, for parents of mildly overweight children, the YHC professional may be the first to point out that the child is overweight [34]. Parents often misperceive the child's weight status or are unaware of the consequences of excess weight for their child [34], [35], [36], [37]. During the visit the YHC professional may therefore motivate parents to change behavior, or at least create awareness.

There are some factors in the intervention implementation and study that may have limited the detection of potential intervention effects. Results showed a lack of attendance to the additional counseling sessions, in line with other studies implementing this type of additional health visits [38], [39], [40]. First, our registration showed that YHC professionals were not able to invite all parents to an additional session, partly due to the unwillingness of parents to attend these sessions. Although previous research has shown the positive effects of a motivational interviewing approach [39], [41] even when working with parents of obese children [38], the YHC professionals reported difficulties in motivating parents to attend additional counseling sessions or changing health-related behavior. In this study, the YHC professionals were provided with a one-day workshop on motivational interviewing. This level of instruction may not be optimal because research has suggested the beneficial effects of refreshment sessions and feedback on performance [38], [42], [43]. Instruments are currently being developed for evaluating motivational interviewing performance [44]. However, the integration of this study in current practice, together with time and budget restraints, made monitoring the YHC professionals' skills and performance with regard to using the prevention protocol unfeasible but an important issue for future implementation of the protocol.

Secondly, the data showed that the YHC professionals often failed to start with the intervention because they considered the child not overweight, even though according to the international cut-off values the child was overweight; this is described in the prevention protocol as the ‘clinical judgment’ [17] [18]. The clinical judgment can change the decision with regard to a child's weight status based on factors potentially influencing the weight status of the child: posture, body composition, ethnicity and other factors [18]. Nevertheless, the researchers instructed and emphasized that the YHC professionals should offer the prevention protocol to all children diagnosed having overweight according to the international cut-off values. Implementation of the intervention may have been less uniform due to the use of the clinical judgment by some YHC professionals. In addition to the clinical judgment or the cut-off values, the lifestyle of parent and child may be the foremost starting point for the decision on whether or not to offer the prevention protocol.

The parents that did attend an additional session had children with higher BMI's, this may have influenced the intervention effect observed as well. The intervention was designed for overweight prevention, including children that only just meet the criteria for overweight, for which it also appeared to be effective. For obese children, more intensive, multidisciplinary interventions seem to be more effective in changing health behaviors [5], [45], [46]. Mechanisms contributing to behavior change, for example self-efficacy and habit strength, [47], [48] [49] may be different between overweight and obese children. In addition, as suggested by Wake and colleagues [50], the effect of the intervention may depend on the willingness of the parents to change, which may be greater among parents of overweight children or parents who self-initiate participation in interventions.

In line with studies performed in the primary care setting minor effects were observed from providing an intervention [6], [38], [40], [51]. But, youth health care with high attendance rates of parents and children at regular appointments throughout the infant, child and adolescent period, during which height and weight measurements are taken, offers opportunities for tailored prevention [12], [11], [13]. As part of a community approach to overweight prevention, the opportunity to intervene in youth health care may not be passed [50]. So, despite the fact that we could not demonstrate a convincing statistically significant effect between the intervention and control condition with regard to BMI or waist circumference, we believe that youth health care may contribute to overweight prevention. Efforts are needed to optimize the protocols that can be implemented in this setting.

Hypothetically, to enhance effects the prevention protocol may best be implemented during the well-child visit; which most parents attend. Therefore, we recommend integrating elements of the prevention protocol in the well-child visit. Not all elements can be integrated and to prevent parents from dropping out before beginning the additional sessions, the first additional session should be planned shortly after the well-child visit [38]. Also, alternatives to face-to-face sessions could be telephone sessions or Internet-tailored advice. More personal contact with parents (e.g., text messages or e-mail) may increase participation and/or support sustained behavioral changes. However, even with optimal implementation of the intervention, an approach in which all health care organizations and both public and private institutions work together to create an overall healthier environment may be essential to effectively address childhood overweight on a societal level [52].

### Strengths and limitations

The strengths of this study include the broad acceptance and use of the prevention protocol in the YHC setting across the Netherlands, the large number of parents participating in the study which enabled the detection of overweight children, and the two-year follow-up. By using standardized protocols for the measurement of height, weight and waist circumference, measurement errors in this respect were kept limited.

Limitations include falling attendance of parents to the additional sessions and possible contamination in the control condition. Limited information is available on the actions undertaken in the control conditions. In the control condition, additional sessions were planned sporadically (data not shown) and a lot of media attention was given to the protocol. Although not according to the prevention protocol, YHC professionals in the control condition provided, usual, tailored care with regard to overweight prevention. Taken together, this may have decreased the potential to detect an effect of the intervention comparing both conditions.

### Conclusion

With the prevention protocol, parents of overweight (not obese) children are offered a low-intensive intervention to change health-related behaviors associated with overweight and obesity. The intervention proved to be effective only among mildly overweight children.

We recommend repeating this study in different settings to confirm the observed results. Further research will need to evaluate adjustments and improvements of the prevention protocol, such as integrating elements in the regular well-child visit, higher parent participation in the additional sessions, and implementation improvement (i.e. training and feedback to the intervention practitioners) on health outcomes. More specifically, child health-related behaviors (playing outside, watching television, having breakfast and drinking sweetened beverages), psychosocial outcomes (psychological well-being, quality of life) and parent health-related behaviors will be evaluated complementarily to the weight-related outcomes.

In conclusion, the prevention protocol is designed to be implemented in practice and is rated positively by practitioners and parents. Overall, in line with McCallum et. al. [40], we emphasize the importance of determining whether and how, in this case, the setting of (school-based) preventive youth health care can contribute in overweight prevention among children.

## Supporting Information

Checklist S1 **CONSORT checklist.**
(PDF)

Figure S1 **Graphical representation of the cluster-corrected regression model.**
(PDF)

Protocol S1 **Trial protocol.** Translated version of the trial protocol originally approved by the ethics committee.
(DOC)

Table S1 **Descriptive characteristics for the study population of the ‘Be active, eat right’ study (n = 8 784).**
(DOC)
