# Supplementary figures and images for: Population-Based Childhood Overweight Prevention: Outcomes of the ‘Be Active, Eat Right’ Study
Source: PLoS One. 2013 May 31;8(5):e65376. doi: 10.1371/journal.pone.0065376 (PMC3669240; doi:10.1371/journal.pone.0065376)

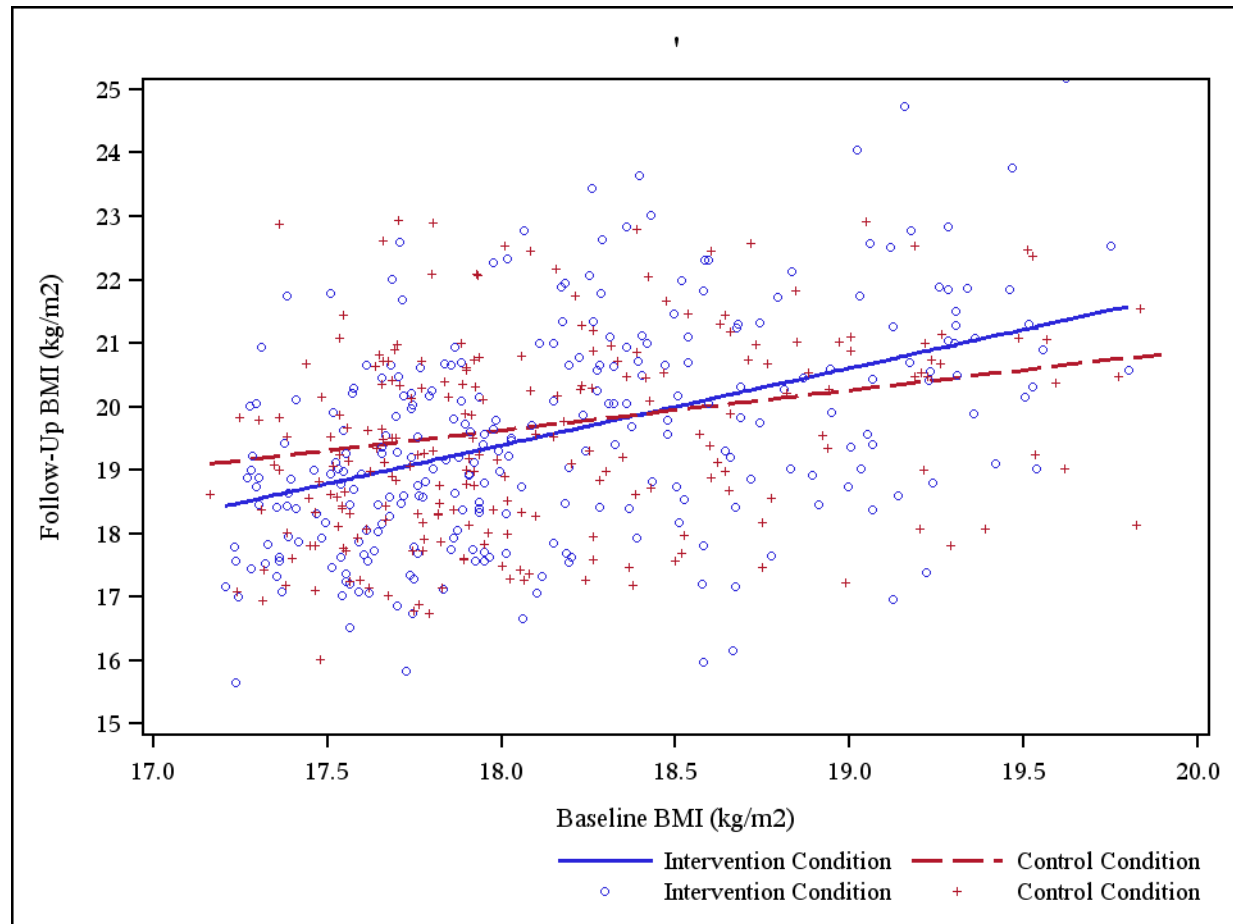

Supplement: Figure S1 — Graphical representation of the cluster-corrected regression model. (PDF) [file pone.0065376.s002.pdf]
