# Supplementary material for: Population-Based Childhood Overweight Prevention: Outcomes of the ‘Be Active, Eat Right’ Study
Source: PLoS One. 2013 May 31;8(5):e65376. doi: 10.1371/journal.pone.0065376 (PMC3669240; doi:10.1371/journal.pone.0065376)
Supplement: Protocol S1 — Trial protocol. Translated version of the trial protocol originally approved by the ethics committee. (DOC) [file pone.0065376.s003.doc]

| **RESEARCH PROTOCOL – translated version** |
| --- |

# “Be active, eat right”: a healthy lifestyle for children 5 to 6 years. Evaluation of the Dutch preventive Youth Health Care (YHC) Overweight Prevention-protocol and Overweight Detection-protocol among 5-year-old children in a cluster randomized controlled trial

| **Project** |  |
| --- | --- |
| **Project financing** | **ZonMw** |
| **Short title** | “Be active, eat right”: a healthy lifestyle for children 5 to 6 years. |
| **Date** | **June 2007** |
| **Projectleader** | Dr H Raat  Department of Public Health  Erasmus MC  P.O. Box 2040  3000 CA Rotterdam, the Netherlands  h.raat@erasmusmc.nl  tel 0031 10 40 88095 |

**SUMMARY**

**Relevance**

The prevalence of overweight and obesity among children has at least doubled in the past 25 years, especially in socially disadvantaged and specific ethnic subgroups. Dutch Youth Health Care (YHC) may contribute to the prevention of overweight and obesity by the recently developed YHC Overweight Detection-protocol (Signaleringsprotocol Overgewicht) and Prevention-protocol (Overbruggingsplan Overgewicht). The Detection-protocol identifies children with “overweight but no obesity”, and these children and their parents are offered the Prevention-protocol with up to four visits with a program of non-directing behavioral counseling to improve health behaviors and to reduce body fatness. Children with “obesity” will be referred to the family physician. Feasibility and acceptability have been established. However, a rigorous effect evaluation is not available yet. Municipal Health Services (MHS), on a broad scale, have already implemented the Detection-protocol. Municipal Health Services are also eager to implement the Prevention-protocol, although no effect evaluation is available yet.

**Aims**

The Erasmus University Medical Center (ErasmusMC) in Rotterdam will together with the Free University Medical Center (VUMC) in Amsterdam evaluate both the Detection-protocol and the Prevention-protocol.

**Study design**

The study design will be a cluster-randomized trial with one intervention condition and one control condition. The YHC-teams will be the unit of randomization within the MHS. Data collection will take place during and before the regular preventive health check (preventief gezondheidsonderzoek - “PGO”) that takes places when the child is around 5 to 6 years old. After one year and two years follow-up measurements will take place.

**Methods**

Thirty-six YHC teams from six Municipal Health Services will invite, during one school year, 14.400 5-year-olds and their parents to participate in the study with an expected participation rate of 50% (n=7.200). Assuming a prevalence of “overweight, not obesity” of 9% and a dropout of maximally 30%, we expect complete data on 450 children with “overweight not obesity at baseline” of whom 225 belong to the Intervention group and 225 to the Control group. In the Intervention group both the Detection-protocol and the Prevention-protocol will be applied, while in the Control group the Detection-protocol will be applied in combination with ““usual care”” (care before the Prevention-protocol). In order to evaluate the Detection-protocol data of around 1075 other children will be included in the study.

**Primary outcomes**

Primary outcomes can be divided in outcomes on behalf of the Prevention-protocol and outcomes on behalf of the Detection-protocol. To evaluate the effects of the Prevention-protocol measures of body fatness and of overweight-reducing and -inducing behaviors (breakfast, sweet beverage consumption, physical activity/ outside play, television viewing/ computer gaming) will be compared among children with overweight who will receive the Prevention-protocol and children in the control condition receiving ““usual care””. The YHC professionals applied labels “being overweight but not obese”, “obese” respectively “normal weight” according to the Detection-protocol in 5-year-olds and these labels will be applied at age 7 years to evaluate the predictive value of the Detection-protocol in the control condition.

**Participant burden**

All children will undergo measures of height, weight and waist circumference. After two years of follow-up these measures will be repeated. In addition parents will fill in questionnaires. With these questionnaires, baseline data is gathered and overweight-related behaviors are investigated. At one and two years follow-up parents will fill in the questionnaires again.

**RELEVANCE**

Overweight is a growing problem. The prevalence of overweight and obesity among children has at least doubled in the past 25 years, especially in socially disadvantaged and specific ethnic subgroups (Fredriks et al, 2000; Hirasing et al, 2001; Fredriks et al, 2005; Whitlock et al, 2005). Body Mass Index (BMI) in childhood tracks to adulthood moderately to very well (Guo et al, 1999; Freedman et al, 2001; Magarey et al, 2003). Adverse health effects of adult obesity, resulting in considerable loss of healthy-life expectancy, have been well documented (Whitlock et al, 2005). Obesity in adults is associated with increased prevalence of diabetes, cardiovascular disease, distinct types of cancer and orthopedic problems (Whitlock et al, 2005). Obesity in childhood has been shown to be associated with increased adult morbidity and mortality independent of adult weight (Must et al, 1999). Adverse health effects of childhood obesity that already emerge during childhood such as diabetes type II, increased levels of cardiovascular risk factors, knee complaints, apnea during sleep and psychosocial problems have also been documented (Bulk-Bunschoten et al, 2005).

Dutch Youth Health Care (YHC) may contribute to the prevention of overweight and obesity by the recently developed YHC Overweight Detection-protocol (Signaleringsprotocol Overgewicht) and Prevention-protocol (Overbruggingsplan Overgewicht). In 2004 the national Overweight-Detection-protocol (Signaleringsprotocol Overgewicht in de Jeugdgezondheidszorg) was developed (Bulk-Bunschoten et al, 2005). This Detection-protocol creates uniform monitoring of overweight in children by YHC-teams and may contribute to epidemiological research and national monitoring. In addition to the Overweight Detection-protocol, an YHC Overweight Prevention-protocol for the subgroup children at risk for the development of obesity (as identified by the YHC Detection-protocol) has been developed (Overbruggingsplan; HiraSing et al, September 2005). In this Prevention-protocol a method for individual primary and secondary prevention for children with overweight is described, for use in the YHC.

Both the detection- and the Prevention-protocol have been tested with regard to feasibility and acceptability (van de Laar et al, 2006). The Detection-protocol is currently, fully or partially, used by YHC teams. Also, the Prevention-protocol is (partly) used by some YHC teams. Both protocols have not been evaluated. Before implementation of the Prevention-protocol in YHC a careful effect evaluation is needed.

**AIMS**

Between 2003-2006 the VUMC conducted the PROMIS-project. Goal of this project was the development and evaluation of a Minimal Intervention Strategy within YHC for secondary prevention of overweight among 5-year old children. The conclusion of the project was that there were opportunities for prevention of overweight in children in the YHC setting. However, with regard to effectiveness of the Minimal Intervention Strategy no conclusions could be given. The advice after the project was to continue with the Prevention-protocol for now in the YHC.

The goal of the collaborative “Be active, eat right” study of the ErasmusMC and the VUMC is performing the evaluation of the Detection-protocol and the Prevention-protocol. For this evaluation 5-6 year old children and their parents will participate. The study consists of multiple parts: evaluation of the Prevention-protocol, evaluation of the Detection-protocol, a process evaluation and cost-effectiveness evaluation. The research question are as follows:

Overweight Prevention-protocol

1. What are the effects of the Overweight Prevention-protocol among children who have been identified as overweight according to the Overweight Detection-protocol in terms of measures of body fatness and in terms of overweight-reducing and -inducing behaviors at 1 and 2 year follow-up?

Overweight Detection-protocol

1. How well do overweight and normal weight defined by the YHC physician according to the Detection-protocol, in the absence of the Prevention-protocol, predict overweight and normal weight at 2 year follow-up, and how is this moderated by the parent-reported overweight-reducing and -inducing behaviors and by participation in weight-management interventions?

Process evaluation

1. What is the adherence of parents/children and YHC professionals to the distinct elements of the Detection- and Prevention-protocols, how do they appreciate these elements, how often and to whom are these elements of the protocols applied, respectively should have been applied, and what are, if any, negative side effects of the interventions in terms of worry, stigmatization, lowered self-esteem, and development of relative underweight?

Cost-effectiveness analysis

1. What is the ratio between costs and effects in terms of relative reduction of measures of body fatness and in terms of modeled future health effects (Quality-adjusted life years) of the Prevention-protocol compared to the “usual care” group?

In this study we will also explore the differences in effects and process characteristics (e.g. adherence) for subgroups socially disadvantaged and non-Dutch children.

**DESIGN**

In figure 1 a schematic display is given of the participants of the study. Participants in the study can be divided in three levels: 1) MHS, 2) YHC-teams (physician, nurse, assistant) and 3) children and their parents.


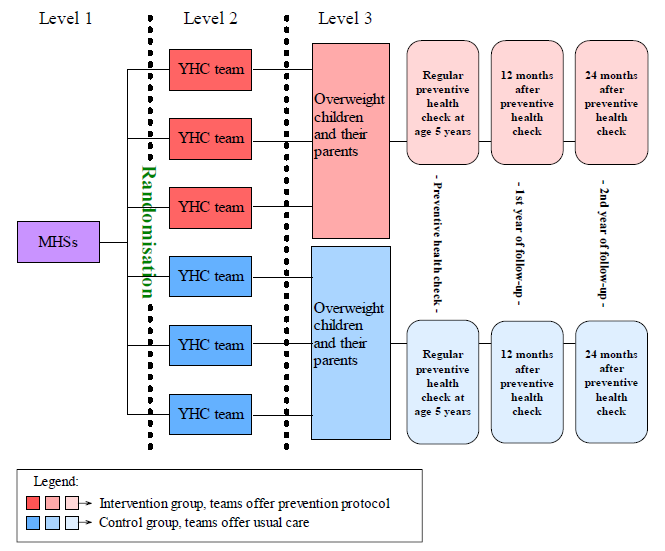


*Figure 1* Systematic presentation of a (imaginary) participating MHS and the YHC-teams and children covered by this MHS.

**Prevention-protocol**

The study design for the effect evaluation of the Prevention-protocol is a cluster-randomized controlled trial (c-RCT) with a follow-up period of 2 years. The level at which participants are clustered within the study is the YHC-team. This is also the level of randomization.

**Detection-protocol**

The design for the evaluation of the Detection-protocol is observational and will be performed in the control group of the c-RCT. All children participating in the study and allocated to the control group will be evaluated according to the Detection-protocol. By using the Detection-protocol children will be labeled having ‘normal weight’ ‘overweight, not obesity’ or ‘obesity’. After 2 years all children will be evaluated using the Detection-protocol again and their weight will be labeled ( ‘normal weight’, ‘overweight, not obesity’ or ‘obesity’). By this approach the predictive value of the Prevention-protocol for predicting weight of children at age 7 years can be evaluated.

**PARTICIPANT NUMBERS**

**Participants**

- According to power calculations at least 36 YHC-teams will need to participate in the study.
- The 36 YHC-teams are expected to invite in total at least 14.400 5 to 6 year old children and their parents for the regular preventive health check (“PGO”) at age 5 between 2007-2008.
- The percentage participants to the study is estimated at 50%; 7200 children.
- During the “PGO”, the Detection-protocol will be used to categorize children in BMI category. The prevalence of ‘overweight, not obesity’ is estimated at 9%. That will be at least 648 children.
- The drop-out rate during the study is estimated at 30%. At 2-year follow-up of 450 children with ‘overweight, not obesity’ measurements will be available.
- Of the children with ‘overweight, not obesity’ one half will be allocated to the intervention group (225 children) and the other half will be allocated to the control group (225 children).
- In the control group the number of children with obesity will be around 38 (all children with obesity in the control group).
- In the control group the number of children with normal weight will be around 1050 (sample of children with normal weight in the control group).
- The total number of participants in the intervention group after two year follow-up will be around 225.
- The total number of participants in the control group after two year follow-up will be around 1300.

**STUDY PLAN**

**Recruitment of MHS’s**

At the start of the research project MHS’s throughout the Netherlands will be recruited to participate, from urban as well as rural areas. The MHS’s that participate in the study have to be representative for all MHS’s in the Netherlands. The distribution, social-economic-position and ethnicity of the children and their parents, must be representative to the Dutch population. This way the results will be better generalized.

The MHS will first receive an information package about the study. This package will contain a letter that shortly explains the study. There will be a project description included describing exactly what is expected from the MHS’s. Finally, the package contains an answering form which the MHS can fill in when they want to participate in the study. In addition, MHS’s will be contacted by phone.

In all participating MHS’s an summary of ‘“usual care”’ that is provided to overweight children and their parents will be created. It is important that the used procedures and protocols are mapped before the start of the study.

Among MHS’s that indicate interest for participating in the study, a inventory of the YHC-team composition (which physician, nurse and assistant) will be made. Also, MHS’s are asked to indicate which of their YHC teams they think are suitable for participation. It is important to have stable YHC teams which, if possible, have a stable team composition, this will contribute to the execution of the study. Instable teams or teams that overlap will be excluded from participation.

Finally, the teams within each MHS will be randomized to control or intervention group. The YHC teams allocated to the control group will perform care as usual, after a child with overweight is detected using the Detection-protocol. The YHC teams allocated to the intervention group will provide care according to the Prevention-protocol when they detect a child with overweight according to the Detection-protocol. The Prevention-protocol describes that three follow-up counseling visits can be offered to parents and children. The YHC-teams are instructed to fully complete the Prevention-protocol. During the counseling visits motivational interviewing is used. The YHC professionals will be receiving a training is correct use of the protocols and motivational interviewing. The training will be in groups.

**Recruitment of children and their parents**

The study population consists of all children and their parents that will be invited for the ““PGO”-5-year-olds” during school year 2007/2008 that belong to one of the participating YHC-teams of the participating MHS’s. All parents of the children in the study population will, circa 2 months before the scheduled “PGO”, be informed about the study. Together with the invitation a informed consent form for the study is provided. Parents are asked to provide written informed consent to participate in the study and take the informed consent form with them to the “PGO”.

The study population consists in principle of all children and their parents that will be invited for the “PGO” at child age 5-year, during school year 2007/2008 that belong to one of the participating YHC-teams of the participating Municipal Health Services. However, for the Prevention-protocol and the questionnaires in the study it is necessary to have basic mastery of the Dutch language. Also, children that do not have weight as a primary problem, but for example physical or mental problems, will visit the YHC. The YHC professional will than decide whether or not these children will participate in the study.

**DATA COLLECTION**

**Baseline**

Prior to the informed consent procedure, parents will be invited to complete a concise one-page questionnaire irrespective of their willingness to participate in one or more parts of the study. The Municipal Health Services will mail the one-page questionnaire and the consent materials along with the regular “PGO” invitation to the parents in their area. This questionnaire will measure socio-demographic items including educational level and ethnic background, key target behaviors relevant for the development of overweight, the attitude concerning the importance of healthy behaviors and prevention of overweight,

We assume that more parents will respond to this questionnaire than will provide informed consent to participate in the study. Information from the questionnaire will be used to evaluate the correlates of providing informed consent to participate in the study and of subsequent dropout from the study. This questionnaire will also provide baseline data before any YHC intervention has taken place, either the Detection-protocol or Prevention-protocol.

**Detection-protocol**

During the “PGO” measurements described in the YHC national work package will be performed (Basistakenpakket JGZ). Among all children participating to the study the Detection-protocol will be applied during the “PGO”. Children’s height and weight will be measured and according to the Detection-protocol children will be labeled ‘normal weight’ ‘overweight, not obesity’ or ‘obesity’. Children with obesity will be referred to the general practitioner. Children with overweight are offered care, in the control condition this will be “usual care”, in the intervention condition the Prevention-protocol will be offered.

**Measurement waist circumference and questionnaire**

Next to the height and weight measurement there will be a measurement of waist circumference. Parents of participating children will be asked to fill in a questionnaire which will collect additional data with regard to:

- overweight related behaviors (breakfast, sweet beverage consumption, physical activity/ outside play, television viewing/ computer gaming)
- health-related quality of life
- indicators of negative side effects of the intervention
- participation in weight management programs

**Intervention group**

In the intervention group care will be provided according to the YHC Prevention-protocol whenever the YHC professional labels a child as “overweight not obese” according to the Detection-protocol. In short, the YHC-physician will assess whether the parents are motivated to participate in counseling to promote overweight preventing behaviors and parents are offered up to three structured lifestyle counseling visits. During the counseling visits motivational interviewing is used by the YHC professional. The counseling visits are targeted at the following four behaviors:

1. having breakfast daily
2. drinking less sweet beverages
3. stimulating daily physical activity and outside play
4. limiting tv viewing and computer activities

Firstly, a short interview will determine which of the four behaviors will be targeted. During the first counseling visit there will be discussion with the parents about the health behaviors. Than will be determined on which of the four behaviors advice is needed. The “PGO” is limited in time and it will not always be possible to fit the first counseling visit in the “PGO”. In these situations follow-up counseling visits can be planned to further discuss the health behaviors. When the parents agree eating and activity dairies can be provided. The YHC professional will ask the parents to fill these in during the period of additional counseling. In total three counseling visits can be offered to parents to provide advice with regard to the four among children overweight related behaviors.

For the process evaluation the intervention group will gather additional data before and during the “PGO”. This data will inform about the use of the Detection-protocol and the Prevention-protocol. In the Intervention group, process characteristics of the Detection-protocol and the Prevention-protocol will be measured additionally by questionnaires for parents who participate in the Prevention-protocol, and by questionnaires for the YHC-professionals that carry out the Prevention-protocol. The questionnaire for the parents will assess: satisfaction with the protocols, whether they understand different elements of the protocol, whether the advice they received with regard to the four behaviors suited them and how much time is needed for the counseling visits. The YHC professionals will register how the “PGO” developed and how the adherence is to both protocols. They will also register how the additional counseling visits develop, whether parents agree on the additional counseling visits and whether parents visit the MHS for the counseling visits.

**Control group**

In the control “usual care” will be offered, care before the Prevention-protocol, to the overweight children and their parents.

**1^st^ follow-up measure**

The first follow-up questionnaire will be send 12 months after the ““PGO””, which is similar to the questionnaire at baseline. If proven feasible, parent will self-report on child waist circumference by an included measuring tape.

**2^nd^ follow-up measure**

After 24 months a second follow-up questionnaire will be send to parents. Biometry, including weight, length, waist circumference will be repeated. Based on weight, length, Body Mass Index, “normal weight”, “overweight not obesity” or “obesity” according to the Detection-protocol will be labeled (children are 7 years).

**OUTCOME MEASURES**

**Baseline measurements**:

- Demographic and general characteristics;
- Data concerning pregnancy, birth weight, breastfeeding and medical history;
- Body mass index, and behaviors of parents themselves;
- Body mass index relative to sex and age specific norms and waist circumference (Cole et al, 2000);
- Presence of overweight reducing and inducing behaviors (specific behaviors targeted in the intervention) (Promis questionnaires; van de Laar et al, 2006);
- Health-related quality of life (CHQ-PF28; Raat et al, 2005);
- Attitudes of parents regarding the specific health behaviors (Promis questionnaires; van de Laar et al, 2006);
- Baseline levels of indicators of negative side effects.

**Evaluation of the Prevention-protocol, primary outcomes:**

- Relative change in Body Mass Index 24 months after the ““PGO”-5-year-olds” in children with “overweight not obesity” in the Intervention group relative to the Control group (Doak et al., 2006; Cole et al, 2000).

**Evaluation of the Prevention-protocol, secondary outcome measures**:

- Presence of label “overweight” (respectively “obesity”) according to Detection-protocol at age 7 (yes/no) (Bulk-Bunschoten et al, 2005);
- Waist circumference (Cole et al, 2000);
- Levels of the five target overweight reducing and inducing behaviors (Promis questionnaires; van de Laar et al, 2006). For example: Change in average daily number of minutes watching TV, video, DVD or playing on a computer, 24 months after the ““PGO”-5-year-olds” in children with “overweight not obesity” in the Intervention group relative to the Control group (Doak et al., 2006; Renders et al., 2004);
- Health-related quality of life (CHQ-PF28; Raat et al, 2005);
- Attitudes of parents regarding the specific health behaviors (Promis questionnaires; van de Laar et al, 2006)
- Absence/presence of indicators of negative side effects (worry, stigmatization, lowered self-esteem, and development of relative underweight) (Doak et al., 2006).

**Evaluation of the Detection-protocol, most important outcomes:**

- Predictive value of the labels “normal weight”, “overweight but not obesity”, and “obesity” at the ““PGO”-5-year-olds” according to the Detection-protocol for the presence of these labels at age 7 years, in the absence of weight-management interventions (Bulk-Bunschoten et al, 2005).
- Sensitivity and specificity of the Detection-protocol at the ““PGO”-5-year-olds” for “overweight but not obesity”, and “obesity” at the age of 7 years, in the absence of weight-management interventions.
- Furthermore it will be assessed in how far this is modified by the five relevant target behaviors and by specific measures of body fatness other than Body Mass Index, such as skin fold measures and waist circumference.

**Measures regarding the Process evaluation:**

- Adherence to the distinct elements of the interventions;
- Satisfaction of the parents with the interventions (modified Patient Satisfaction questionnaire) (Martinali et al, 2001);
- Understandability of program elements for parents (Promis questionnaire, van de Laar et al, 2006);
- Rating of possibility for application in day-to-day life by parents (van de Laar et al., 2006);
- Time investments of parents and YHC-professionals for YHC- visits;
- Acceptability for the YHC-professionals of the interventions (van de Laar et al., 2006).

**Measures regarding the Cost-effectiveness evaluation:**

- the costs of counseling visits,
- education of YHC-personnel,
- other program costs (e.g. non-client related time investments of YHC-personnel with respect to program),
- time and travel costs of parents and children.

**Materials**

The following measures will be used during the study (baseline, performing the Detection-protocol, care trajectory after detecting overweight (Prevention-protocol versus “usual care”), follow-up measures, process measures, cost-effectiveness measures):

- questionnaires for parents and YHC professionals
- Detection-protocol and Prevention-protocol as working protocols
- activity and eating diaries to be completed by parents
- materials to determine height, weight and waist circumference: height measure, scale, centimeter tape for waist circumference

For the development of the questionnaires, diaries and other materials, the in the PROMIS project developed materials will be used. In addition, data from children and parents routinely obtained by YHC professionals and further administrative data of the MHS’s will be used.

**STATISTICAL ANALYSES
Effect evaluation of the Prevention-protocol:**

Intention to treat analysis will be applied (Hollis et al., 1999). This will be supplemented secondarily with a per protocol analysis (effects in the subgroup that sufficiently complied with the trial’s protocol) with careful description of correlates of non-adherence and dropout (Heritier et al., 2003).

Multi-level analyses will be applied in order to allow for dependency between the individual measurements within the 36 randomized YHC-teams (Campbell et al, 2004). Multi-level linear regression analyses will be applied for continuous outcomes and multi-level hierarchical logistic regressions for dichotomous outcomes. Biometric and behavioral outcomes at age 7 years will be analyzed with independent variables: Intervention/Control group, sex, age, normal/low birth weight, breastfeeding, socio-economic status, ethnicity, overweight parents, and baseline levels of the outcome variables. Interaction effects of sex, social disadvantage and ethnic background with the Intervention effect will be explored.

**Evaluation of the Detection-protocol:**

Multilevel analyses or random coefficient analyses will be applied to correct for nested data in assessing the level of tracking in the Control group of the presence/absence of “overweight not obesity” (and relative body mass index and other biometric measures) from age 5 to age 7 years (Baron et al, 1986; Twisk, 2003). In the models we will explore the mediating and moderating influence of the afore mentioned specific obesity-related relevant behaviors, the moderation of reported participation in weight management or weight-loss interventions, as well as interaction effects of sex, social disadvantage and ethnic background.

**Process evaluation and Cost-effectiveness evaluation:**

The adherence to the interventions by parents and professionals will be described and compared between Intervention/Control group by multiple linear or logistic regression analysis (depending on outcome variable type). Satisfaction with and process characteristics of the interventions (parents/professionals) will be described carefully. Also a cost-effectiveness evaluation will take place.

TIME SCHEDULE AND PLANNING

| Task Planning |  |
| --- | --- |
| 1. Recruitment MHS’s | February- March 2007 |
| 2. METC request | May 2007 |
| 3. Randomization YHC-teams | May 2007 |
| 4. Training day of YHC-teams in using the Detection-protocol (control group and intervention group) and Prevention-protocol (intervention group) | June – September 2007 |
| 5. Develop study materials | April – June 2007 |
| 6. Finalize study materials | July 2007 |
| 7. Examine YHC-teams | August 2007 |
| 8. Meeting to explain YHC-teams procedures | August 2007 |
| 9. Invitation through MHS to parents & children for “PGO” + informed consent + baseline questionnaire | August 2007 – May 2008 |
| 10. During “PGO”:  Detection-protocol (all children that participate in the study) + definition length, weight and means outline (intervention group: about 225 children, control group: about 1300 children) + questionnaire (intervention group: about 225 children, control group: about 1300 children) + Prevention-protocol (intervention group: about 225 children) or “usual care” (control group: about 225 children) | September 2007 – June 2008 |
| 11. 3 continuation counseling visits belonging to Prevention-protocol (intervention group: about 225 children) : 1st counseling visit about 1 month after “PGO” / 2nd counseling visit about 3 months after “PGO” / 3rd counseling visit  about 6 months after “PGO” | October 2007 – November 2008 |
| 12. 1st follow-up measurement, about 1 year after “PGO”: repetition questionnaire (intervention group: about 225 children, control group: about 1300 children) + (when feasibly) self-report waist circumference | September 2008 – June 2009 |
| 13. 2nd follow-up measurement, about 2 year after “PGO”: repetition questionnaire (intervention group: about 225 children, control group: about 1300 children) + repetition Detection-protocol (control group: about 1300 children) + repetition definition length, weight and waist circumference (intervention group: about 225 children,  control group: about 1300 children) | September 2009 – June 2010 |
| 14. Trial evaluation and cost-effectiveness analyses | September 2007 – June 2010 |
| 15. Analysis collected data | June 2009 – September 2010 |
| 16. Report collected data | October 2010 – December 2010 |
| 17. Contribution to implementation of results: closing expert meeting | December 2010 |

**REFERENCES**

Basistakenpakket Jeugdgezondheidszorg 0-19 jaar: Ministerie van Volksgezondheid, Welzijn en Sport; 2002.

Baron RM, Kenny DA 1986 The Moderator-Mediator Variable Distinction in Social Psychological Research: Conceptual, Strategic, and Statistical Considerations. Journal of Personality and Social Psychology 51:1173-1182.

Braet C, Van Winckel M. Long-term follow-up of a cognitive behavioral treatment program for obese children. Behavior Therapy 2000;31:55-77.

Bronnum-Hansen H. How good is the Prevent model for estimating the health benefits of prevention? J Epidemiol Community Health. 1999;53:300-5.

Bulk-Bunschoten AMW, Renders CM, Van Leerdam FJM, HiraSing RA. Detection-protocol overgewicht in de Jeugdgezondheidszorg. Woerden: Platform Jeugdgezondheidszorg; 2005.

Campbell, M.K., D.R. Elbourne, and D.G. Altman, Consort statement: extension to cluster randomised trials. BMJ, 2004. 328: p. 702-708.

Cole TJ, Bellizzi MC, Flegal KM, Dietz WH. Establishing a standard definition for child overweight and obesity worldwide: international survey. BMJ 2000;320(7244):1240-3.

Doak, CM, Visscher, TLS, Renders, CM, Seidell, JC, The prevention of overweight and obesity in children and adolescents: a review of interventions and programs. Obes Rev 2006;7(1):111-36.

Fredriks AM, van Buuren S, Burgmeijer RJ, Meulmeester JF, Beuker RJ, Brugman E, et al. Continuing positive secular growth change in The Netherlands 1955-1997. Pediatr Res 2000;47(3):316-23.

Fredriks AM, Van Buuren S, Sing RA, Wit JM, Verloove-Vanhorick SP. Alarming prevalences of overweight and obesity for children of Turkish, Moroccan and Dutch origin in The Netherlands according to international standards. Acta Paediatr 2005;94(4):496-8.

Fredriks AM, van Buuren S, Wit JM, Verloove-Vanhorick SP. Body index measurements in 1996-7 compared with 1980. Arch Dis Child 2000;82(2):107-12.

Freedman DS, Khan LK, Dietz WH, Srinivasan SR, Berenson GS. Relationship of childhood obesity to coronary heart disease risk factors in adulthood: the Bogalusa Heart Study. Pediatrics 2001;108(3):712-8.

Gortmaker SL, Peterson K et al., Reducing obesity via a school-based interdisciplinary intervention among youth. Arch Pediatr Adolesc Med 1999; 153:409-418.

Gunning-Schepers L. The health benefits of prevention: a simulation approach. Health Policy. 1989;12:1-255.

Guo SS, Chumlea WC. Tracking of body mass index in children in relation to overweight in adulthood. Am J Clin Nutr 1999;70(1):145S-8S.

Heritier SR, Gebski VJ, Keech AC. Inclusion of patients in clinical trial analysis: the intention-to-treat principle. Med J Aust 2003;179(8):438-40.

Hirasing RA, Fredriks AM, van Buuren S, Verloove-Vanhorick SP, Wit JM. Increased prevalence of overweight and obesity in Dutch children, and the detection of overweight and obesity using international criteria and new reference diagrams. Ned Tijdschr Geneeskd 2001;145(27):1303-8.

Hirasing RA, Bulk-Bunschoten AMW, Renders CM. Youth Health Care Overweight-prevention-protocol (Het Prevention-protocol voor kinderen met overgewicht). Amsterdam: Free University Medical Center; 2005.

Hollis S, Campbell F. What is meant by intention to treat analysis? Survey of published randomised controlled trials. Bmj 1999;319(7211):670-4.

Lobstein T, Baur LA. Policies to prevent childhood obesity in the European Union. Eur J Public Health 2005.

Magarey AM, Daniels LA, Boulton TJ, Cockington RA. Predicting obesity in early adulthood from childhood and parental obesity. Int J Obes Relat Metab Disord 2003;27(4):505-13.

Martinali, J., C. Bolman, et al. A checklist to improve patient education in a cardiology outpatient setting." Patient Educ Couns 2001; **42** (3): 231-8.

Mok E, Beghin L, Gachon P, Daubrosse C, Fontan JE, Cuisset JM, et al. Estimating body composition in children with Duchenne muscular dystrophy: comparison of bioelectrical impedance analysis and skinfold-thickness measurement. Am J Clin Nutr 2006;83(1):65-9.

Must A, Strauss RS. Risks and consequences of childhood and adolescent obesity. Int J Obes Relat Metab Disord 1999;23 Suppl 2:S2-11.

Raat H, Botterweck AM, Landgraf JM, Hoogeveen WC, Essink-Bot ML. Reliability and validity of the short form of the child health questionnaire for parents (CHQ-PF28) in large random school based and general population samples. J Epidemiol Community Health 2005;59(1):75-82.

RIVM/VTV. Population Health Forecasts 2006. Bilthoven: RIVM, forthcoming 2006.

Robinson TN, Reducing children’s television viewing to prevent obesity; a randomized controlled trial. JAMA, 1999; 282; 16: 1561-1567.

Rollnick S., Butler, CC, McCambridge J et al., Counseling visits about changing behaviour. BMJ 2005; 331; 961-963.

Tennefors C, Forsum E. Assessment of body fatness in young children using the skinfold technique and BMI vs body water dilution. Eur J Clin Nutr 2004;58(3):541-7.

Twisk JWR 2003 Applied longitudinal data analysis for epidemiology: a practical guide, 1st ed. Cambridge University Press.

Van de Laar CWE, Renders CM, HiraSing RA, Preventie van overgewicht: een minimale interventie strategie bij 5/6 jarige kinderen binnen de YHC. Presentatie bij NWO-werkgemeenschap Jeugd&Gezondheid. 20-21 januari 2006.

Veerman JL, Barendregt JJ, Mackenbach JP. The European Common Agricultural Policy on fruits and vegetables: exploring potential health gain from reform. Eur J Public Health. September 2005.

Whitlock EP, Williams SB, Gold R, Smith PR, Shipman SA. Screening and interventions for childhood overweight: a summary of evidence for the US Preventive Services Task Force. Pediatrics 2005;116(1):e125-44.
