# Supplementary material for: Population-Based Childhood Overweight Prevention: Outcomes of the ‘Be Active, Eat Right’ Study
Source: PLoS One. 2013 May 31;8(5):e65376. doi: 10.1371/journal.pone.0065376 (PMC3669240; doi:10.1371/journal.pone.0065376)
Supplement: Table S1 — Descriptive characteristics for the study population of the ‘Be active, eat right’ study (n = 8 784). (DOC) [file pone.0065376.s004.doc]

**SUPPLEMENT**

**Table S1** Descriptive characteristics for the study population of the ‘Be active, eat right’ study (n=8 784)

|  | Overall  (n=8 784) | Intervention condition  (n=4 842) | Control condition  (n=3 942) | *p-value** | *p-value*** |
| --- | --- | --- | --- | --- | --- |
| **Child characteristics** |  |  |  |  |  |
| Age, mean (sd), months (missing n=38) | 68.93 (5.07) | 68.86 (5.09) | 69.03 (5.06) | 0.121 | 0.776 |
| Gender (% boys) (missing n=76) | 50.9 | 51.7 | 50.0 | 0.055 | 0.106 |
| Ethnicity (% Dutch) (missing n=176) | 84.9 | 83.9 | 86.2 | **0.002** | 0.627 |
| BMI, mean (sd), kg/m² (missing n=34) | 15.49 (1.52) | 15.53 (1.49) | 15.43 (1.54) | **0.002** | 0.158 |
|  |  |  |  |  |  |
| **Mothers’ characteristics** |  |  |  |  |  |
| Age, mean (sd), years (missing n=1 116) | 36.30 (4.46) | 36.31 (4.50) | 36.28 (4.41) | 0.786 | 0.939 |
| Country of birth (% the Netherlands) (missing n=108) | 89.4 | 88.8 | 90.1 | **0.023** | 0.588 |
| Education level (missing n=167) |  |  |  | **0.046** | 0.508 |
| Low / Mid-low | 23.6 | 24.3 | 22.8 |  |  |
| Mid-high/ High | 76.4 | 75.7 | 77.2 |  |  |
| BMI categories (missing n=921) |  |  |  | **0.018** | 0.264 |
| Normal weight | 70.1 | 69.1 | 71.3 |  |  |
| Overweight/ obesity | 29.9 | 30.9 | 28.7 |  |  |

* P-value derived from Chi-Square tests comparing intervention and control condition on categorical and binomial outcomes, p-value derived from independent samples t-test comparing intervention and control condition on continuous outcomes.

** P-value derived from multilevel regression model, demographic characteristic predicted by research condition (reference control condition). For example, there is no difference in age (p=0.776) between the children in the intervention clusters and control clusters.

Note: bold numbers indicate significant p-value
